# In and Out of the Bursa—The Role of CXCR4 in Chicken B Cell Development

**DOI:** 10.3389/fimmu.2020.01468

**Published:** 2020-07-14

**Authors:** Nandor Nagy, Florian Busalt, Viktoria Halasy, Marina Kohn, Stefan Schmieder, Nora Fejszak, Bernd Kaspers, Sonja Härtle

**Affiliations:** ^1^Department of Anatomy, Histology and Embryology, Faculty of Medicine, Semmelweis University, Budapest, Hungary; ^2^Department of Veterinary Sciences, Ludwig-Maximilians-Universität München, Munich, Germany

**Keywords:** chicken, Bursa fabricii, B cell development, CXCR4, cell migration, follicle cortex, follicle medulla, CXCL12

## Abstract

In contrast to mammals, early B cell differentiation and diversification of the antibody repertoire in chickens do not take place in the bone marrow but in a specialized gut associated lymphoid tissue (GALT), the bursa of Fabricius. During embryonic development, B cell precursors migrate to the bursa anlage, where they proliferate and diversify their B cell receptor repertoire. Around hatch these diversified B cells start to emigrate from the bursa of Fabricius and populate peripheral lymphoid organs, but very little is known how the migratory processes are regulated. As CXCL12 (syn. SDF-1) and CXCR4 were shown to be essential for the control of B cell migration during the development of lymphoid tissues in mammals, we analyzed expression and function of this chemokine/chemokine-receptor pair in the chicken bursa. We found a strong variation of mRNA abundance of CXCL12 and CXCR4 in different stages of bursa development, with high abundance of CXCL12 mRNA in the bursa anlage at embryonic day 10 (ED10). *In situ* hybridization demonstrated disseminated CXCL12 expression in the early bursa anlage, which condensed in the developing follicles and was mainly restricted to the follicle cortex post-hatch. Flow cytometric analysis detected CXCR4 protein already on early B cell stages, increasing during bursal development. Post-hatch, a subpopulation with the hallmarks of emigrating B cells became detectable, which had lower CXCR4 expression, suggesting that downregulation of CXCR4 is necessary to leave the CXCL12-high bursal environment. *In vivo* blockade of CXCR4 using AMD3100 at the time of B cell precursor immigration strongly inhibited follicle development, demonstrating that CXCL12 attracts pre-bursal B cells into the bursal anlage. Altogether, we show that CXCL12 and its receptor CXCR4 are important for both populating the bursa with B cells and emigration of mature B cells into the periphery post hatch, and that CXCR4 function in primary B cell organs is conserved between mammals and birds.

## Introduction

The widely accepted dogma is that development of B cells with a highly diversified receptor repertoire is a continuous lifelong process, which takes place in the bone marrow (1). This certainly is true for human and mouse, but many higher vertebrate species use a different strategy to diversify the B cell repertoire, which includes gut-associated lymphoid tissue (GALT) structures (2–4). The prime example for this alternative is the chicken with its primary B cell organ, the bursa of Fabricius (5).

B cell development in the chicken can be broken down in several distinct stages, known as pre-bursal, bursal, and post-bursal phases (6). During the pre-bursal phase, hematopoietic precursor cells, which derive from the intra-embryonic mesenchyme in para-aortic foci, commit to the B cell lineage and spread via the blood to various lymphoid organs like spleen and bone marrow (7). The bursal phase starts between embryonic day 9 (ED9) and ED12, when a small number of pre-bursal stem cells migrates to the bursa anlage, where each of 10,000–12,000 lymphoid follicles is colonized by only 2–5 B cell precursors (7). Within the follicles, cells undergo proliferative expansion (8). In order to recognize the huge magnitude of possible antigens, B cells have to generate a large repertoire of different B cell receptors (BCRs). Therefore, from ED15 onwards, the bursal environment triggers the diversification of the BCR by gene conversion, rather than by gene rearrangement as in human and mouse [reviewed in (9)]. The post-bursal phase begins around hatch and continues until bursa involution. Immature bursal B cells with functional and diversified BCR start to emigrate to the periphery and distribute to the secondary lymphoid organs, where they can encounter antigen and differentiate into antibody secreting cells (10, 11). However, the fraction of emigrating cells is very small and more than 90% of bursal B-cells die by apoptosis (12). Post-hatch, the formerly homogeneous bursa follicles compartmentalize into dense highly proliferating B-cells in a cortex and a more heterogeneous medulla with B-cells and several types of myeloid cells and stroma cells, including bursal secretory dendritic cells (13).

In order to obtain first proliferating and then compartmentalized B cell follicles in the bursa and to seed peripheral lymphatic structures with B cells from the bursa, highly specific sequential cell migration processes are necessary. In a first migration step, B cell precursors have to immigrate into the mesenchyme of the bursal anlage. Then in a second migration step, those earliest bursal B cells have to migrate from the bursal mesenchyme into developing follicle buds. Formation of these follicle buds is triggered by immigrating precursors of bursal secretory dendritic cells (BSDC), which are of hematopoietic origin and cross the basement membrane under the surface epithelium to induce a so-called dendro-epithelial tissue (13, 14). In a third migration step, B cells migrate to the follicle border, when the formerly homogeneous follicles separate into cortex and medulla. Therefore, around hatch, some B cells cross the border and migrate into a layer of desmin and vimentin positive mesenchymal reticular cells, where they continue to proliferate (15, 16). Finally, in a fourth migration step, a small fraction of B cells emigrates mainly from the follicular cortex of the bursa to the peripheral lymphoid organs (17).

Though the different steps of B cell migration are well-described in the literature, very little is known about the inducing and regulating factors. Masteller and colleagues demonstrated that changes in cell-surface glycosylation might be involved in bursa colonization. Pre-bursal and early bursal B cells express the carbohydrate epitope sialyl Lewis(x) (CD15s), a carbohydrate moiety involved in white blood cell adhesion to endothelial cells via selectin binding. In contrast, B cells in bursal follicles, which undergo gene conversion, switch to the high level expression of Lewis(x) (CD15) (18). Interestingly, loss of CD15s around ED15 correlates with the time when developing B cells lose their ability to colonize a bursa (19). Around hatch, a second change in surface glycosylation occurs and B cells downregulate CD15 to low to moderate expression. As peripheral B cells are also CD15-low, it seems likely that preferentially CD15-low cells can leave the bursa (20). Little is known about the expression of selectin E, P, and O or other CD15/CD15s receptors in the bursa, but the reported time-restricted expression pattern strongly suggests a contribution of these molecules to bursal immigration and emigration.

A protein family well-known for inducing cell migration in all vertebrates is the family of chemokines. Among the homeostatic chemokines, which control migration during tissue maintenance and development, is CXCL12 (SDF-1), which interacts with the receptor CXCR4. It was shown that CXCR4-CXCL12 interaction plays an essential role for B cell migration in human and mouse as it regulates stem cell migration into the bone marrow, emigration of immature B cells from the bone marrow (21, 22), migration of germinal center B cells between dark and light zone (23) and the migration of differentiated plasma cells back to the bone marrow (24). Recently, CXCL12 (25) and CXCR4 (26) were also described in the chicken. Using the chick embryo as model system, it was shown that CXCR4 is involved in shape forming processes during ontogenesis, for instance CXCL12-CXCR4 interaction regulates the migration of cardiac neural crest cells and hence contributes to heart morphogenesis (27). Inhibition of CXCR4 signaling reduces immigration of myogenic and angiogenic cells in developing limbs (28), and both physiological development of cloacal musculature (28) as well as the migration of primordial germ cells (29) are determined by CXCL12 dependent chemotaxis.

The aim of this work was to analyze whether CXCR4-CXCL12 interaction does play a role in chicken B cell development in the bursa of Fabricius and thus to address the question whether, beyond the described functions in mammalian bone marrow and peripheral lymphoid structures, this chemokine-chemokine-receptor pair is also important for B cell development in a GALT organ.

## Materials and Methods

### Animals

Fertilized eggs of M11 (B2/B2) chickens were kindly provided by Dr. S. Weigend (Federal Research Institute for Animal Health, Mariensee) and hatched at the Faculty for Veterinary Medicine, Munich. Birds were vaccinated against Marek's disease virus after hatch, housed under conventional conditions in aviaries with groups up to 10 birds, and received food and water *ad-libitum*. Fertilized White Leghorn chicken eggs for *in situ* hybridizations and CAM transplants were obtained from from Biovo Ltd, Hungary. Embryos were staged according to the number of embryonic days (ED). Transgenic green fluorescent protein (GFP)-expressing chicken eggs were provided by courtesy of Prof. Helen Sang and Dr. Adam Balic, The Roslin Institute, University of Edinburgh (30). All animal work was conducted according to relevant national and international guidelines.

### Chorioallantoic Membrane Transplants

Chorioallantoic membrane (CAM) grafts were performed as recently described (31). Briefly, bursa of Fabricius was dissected from ED9 embryos and transplanted on the CAM of ED9 chick. For CXCR4 signaling blocking experiments, the isolated bursa primordium was removed and 1 μl of 200 μM AMD3100 (Sigma Aldrich, St. Louis, USA) was injected into the bursa mesenchymal wall. Then the bursa primordia were cultured on the CAM of GFP-transgenic chickens for 9 days (*n* = 9). PBS used as solvent in the experimental samples was injected to control bursa CAM grafts (*n* = 6).

### Cells

DT40 cells were cultured in IMDM (Biochrom, Berlin, Germany) with 10% FBS, 1% chicken serum (ThermoFisher Scientific, Waltham, USA) and 1 mM ß-mercaptoethanol at 37°C.

Cell suspensions from spleen and bursa were obtained by dissociation of the organs using a 1 ml syringe for embryonic organs or a stainless-steel sieve post-hatch. Leukocytes from spleen, bursa, and blood were then obtained by density gradient centrifugation on Biocoll (1.077 g/ml, Biochrom, Berlin, Germany) as previously described (32).

### RNA Isolation and Quantitative RT-PCR

Pools of bursas or spleens (ED10) or single organs were collected in RNAlater (Merck, Darmstadt, Germany) and stored at −20°C until further processing. Tissues samples were transferred to peqGold TriFast (VWR, Radnor, USA) and homogenized with a tissue homogenizer (Precellys 24, VWR, Radnor USA). Total RNA was isolated according to the manufacturer's Trizol protocol. Quantity and purity of extracted RNA was determined with a NanoDrop 1000 (VWR, Radnor, USA), and the RNA quality was determined using a 2100 Bioanalyzer^®^ (Agilent, Santa Clara, USA). Only RNA samples with an RNA integrity number (RIN) exceeding seven were used for qRT-PCR and microarray analysis.

For cDNA synthesis, genomic DNA was eliminated by DNase I digestion (ThermoFisher Scientific, Waltham, USA) and 400 ng cDNA were generated using the GOScript Reverse Transcription System (Promega Corporation, Madison, USA) according to the manufacturer's instructions. 10 ng cDNA were analyzed for the relative abundance of 18S, CXCR4, and CXCL12 RNA with a GoTaq qPCR Master Mix (Promega Corporation, Madison, USA). Primers for qRT-PCR were designed using PerlPrimer software and obtained from Eurofins, Luxemburg. The following forward and reverse primers were used for qRT-PCR reactions: 18S rRNA: forward primer 5′-CATGTCTAAGTACACACGGGCGGTA-3′ and reverse primer 5-′GGCGCTCGTCGGCATGTATTA-3′, CXCR4 forward primer 5′- CTGTGGCTGACCTCCTCTTTG-3′ and reverse primer 5′- ACACAGGACATTTCCGAAGTACC-3′ and CXCL12 forward primer 5′- CTCAAGAGCAACAGCAAGCAA-3′ and reverse primer 5′- GCCCTTAACGTTCTACCCTTGA-3′. Quantitative RT-PCR was performed using a 7300 Real-Time PCR System^®^ (Applied Biosystems, Warrington, UK) with SYBR-green. Obtained CT values were normalized to 18S rRNA (= dCT) and fold changes (FC) were calculated in comparison to the control group (2^−ΔΔCT^ method).

### Immunohistochemistry

For cryosections, tissue was fixed in 4% formaldehyde for 1 h, then infiltrated with 15% sucrose overnight at 4°C followed by 7.5% gelatin (Sigma Aldrich, St. Louis, USA) in 15% sucrose for 1 h at 37°C, then rapidly frozen at −50°C in isopentane (Sigma Aldrich, St. Louis, USA). Twelve micron-thick cryosections were stained using the primary antibodies listed in Supplementary Table 1 for 45 min, followed by biotinylated goat anti-mouse IgG (Vector Labs, Burlingame, CA, USA) and avidin-biotinylated peroxidase complex (Vectastain Elite ABC kit, Vector Labs). Endogenous peroxidase activity was quenched with 3% hydrogen peroxide (Sigma) for 10 min. The binding sites of the primary antibodies were visualized by 4-chloro-1-naphthol (Sigma Aldrich, St. Louis, USA). Fluorescent secondary antibodies included Alexa Fluor 594 (goat anti-mouse IgG2a and IgG1) and Alexa Fluor 488 (goat anti-mouse IgG2a and IgG1). All Alexa conjugated secondary antibodies were obtained from ThermoFisher Scientific (1:200).

### *In situ* Hybridization

*In situ* hybridization was performed for chick CXCR4 and CXCL12 on paraffin sections using digoxigenin-labeled riboprobes [plasmids were kindly provided by Dr. Beate Brand-Saberi, Ruhr-Universität Bochum, Germany; (33, 34)]. Riboprobe synthesis and *in situ* hybridization were performed according to standard protocols (35).

### Flow Cytometry and Cell Sorting

Staining of cells for flow cytometric analysis was performed according to standard procedures. For two-color immunofluorescence staining, cells were stained with mouse-anti-chB6 and mouse anti-chicken-CXCR4 followed by isotype specific secondary antibodies. For three color staining, cells were stained with mouse anti-chicken-CXCR4 and mouse-anti-chicken-L chain, with mouse-anti-chicken-MHC class II or with mouse-anti-OV-antigen followed by fluorochrome-conjugated isotype-specific secondary antibodies. Then samples were blocked with normal mouse serum (1:20, 20 min) and stained with mouse-anti-chB6 coupled to Alexa-Fluor-647. Detailed information on antibodies is provided in Supplementary Table 1.

Flow cytometry was performed with a FACSCanto (Becton Dickinson, Heidelberg, Germany) and data were analyzed using FACSDiva (Becton Dickinson, Heidelberg, Germany) and FlowJo (FlowJo LLC, Oregon, USA) software.

For the purification of B cells, cell suspensions were stained with mouse-anti-chB6 coupled to Alexa-Fluor-647 and sort-purified using a FACSAriaIIIu with FACSDiva software to a purity of at least 96%.

### Calcium-Flux Assay

One million DT40 cells per sample (>80% viable cells as determined by Trypan blue exclusion) in RPMI with Glutamax, 15 mM Hepes (both ThermoFisher Scientific, Waltham, USA) and 5% FBS (Biochrom, Berlin, Germany) were labeled with 1 ml of 2.5 μM Fluo-4-AM (ThermoFisher Scientific, Waltham, USA) for 30 min at room temperature according to manufacturer's instructions. To examine changes in intracellular calcium levels by flow cytometry, baseline fluorescence was recorded for 60 s before cells were stimulated with ionomycin (0.5 μg/ml, ThermoFisher Scientific, Waltham, USA), anti-chgM (10 μg/ml, Supplementary Table 1), or huCXCL12a (100 ng/ml, ImmunoTools GmbH, Friesoythe, Germany). It was demonstrated that huCXCL12 shows 73% amino acid homology with chicken CXCL12 (25) and acts as agonist for chCXCR4 (26).

To test potential CXCR4 inhibitors, AMD3100 (40 μM, Merck, Darmstadt, Germany), or anti-chCXCR4 (10 μg/ml; Supplementary Table 1) were added first followed by huCXCL12a (100 ng/ml) 30 s later.

### Chemotaxis Assay

Migration assays were performed with either DT40 cells or leukocytes isolated from bursa, spleen and PBL. Assays were carried out at room temperature with endotoxin-free single-use material. 24-well-Transwell^®^ Permeable Supports (Corning, New York, USA) with a pore size of 5 μm were coated with bovine fibronectin (10 μg/ml) (ThermoFisher Scientific, Waltham, USA) dissolved in endotoxin-free distilled H_2_O (Merck, Darmstadt, Germany) and incubated for 1 h at 37°C and 5% CO_2_. Plates were air-dried at 37°C for 2 h. huCXCL12a (ImmunoTools GmbH, Friesoythe, Germany) and AMD3100 (40 μM, Merck, Darmstadt, Germany) or anti-chCXCR4 (10 μg/ml; Supplementary Table 1) were diluted in 600 μl of chemotaxis medium [RPMI containing 0.5% bovine serum albumin (Merck, Darmstadt, Germany)]/well and added to the multiwell plate. Chemotaxis medium served as a negative (chemokinesis) control. Cells were washed twice in warm RPMI (ThermoFisher Scientific, Waltham, USA) and 1 × 10^5^ cells/well in 100 μl of chemotaxis medium were seeded in the plate insert.

After a migration time of 90 min, DT40 cells were taken from the lower chamber, transferred directly into FACS Trucount^®^ tubes (Becton Dickinson, Heidelberg, Germany) and the number of cells was determined by flow cytometry and analyzed with FlowJo (FlowJo LLC, Oregon, USA) software. Migrated primary cells were stained with anti-chB6-AlexaFluor647 (see Supplementary Table 1) before transfer into FACS Trucount tubes.

### ^3^[H] Thymidine Assay

For cell proliferation assays, 1 × 10^6^ bursal lymphocytes per well in RPMI with Glutamax supplemented with 2% chicken serum (ThermoFisher Scientific, Waltham, USA), 8% FBS, 100 IU/ml penicillin and 100 μg/ml streptomycin (all Biochrom, Berlin, Germany) were cultured in 96-well-plates with 100 ng/ml of huCXCL12a in the presence or absence of 5% concentrated supernatants from HEK293 cells, stably transfected with chBAFF or chCD40L. Medium served as control. After 24 h cells were pulsed with [^3^H]-thymidine and harvested 16 h later.

### Statistical Analysis

Statistical analysis (Student's *t*-test, ANOVA) was performed with GraphPad Prism (GraphPad Software, La Jolla, USA). *P*-values were considered significant ^*^ at *p* ≤ 0.05, ^**^ at *p* ≤ 0.01, and ^***^ at *p* ≤ 0.001. Error bars represent the standard deviation for the respective set of samples.

## Results

### CXCL12 and CXCR4 mRNA Expression Vary During Bursa Ontogeny

**Figure 1:**
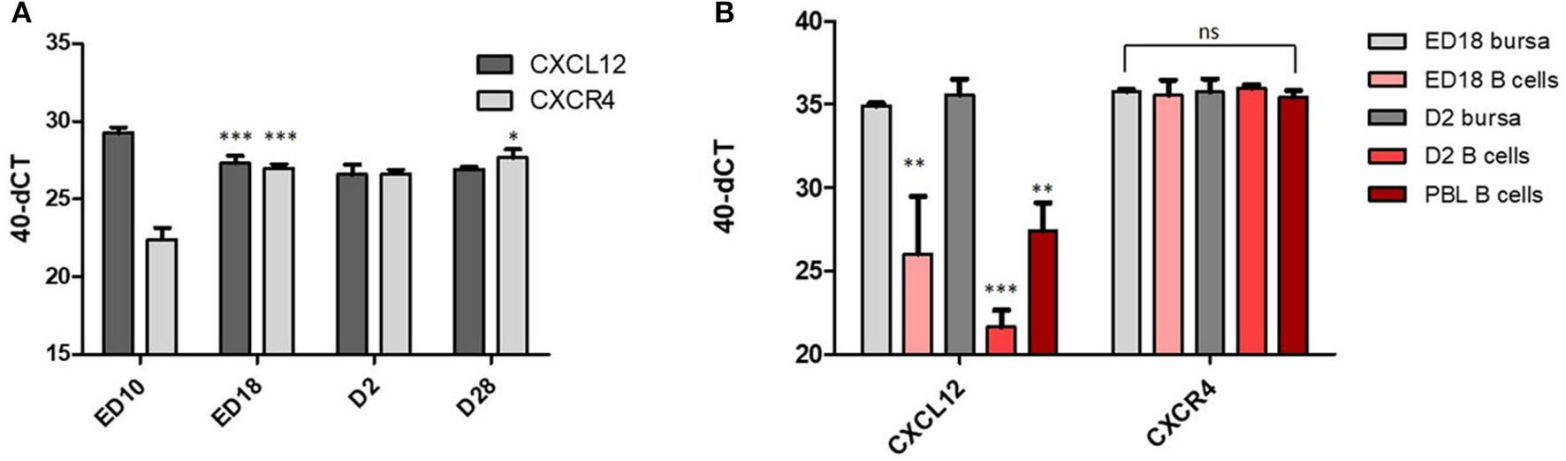
CXCR4 and CXCL12 expression vary during bursa development. **(A)** At the indicated time points, RNA from total bursa (organ pools at ED10, individual organs all other time points; *n* = 5) was analyzed for CXCL12 and CXCR4 expression by qRT-PCR; **(B)** B cells were sort purified from ED18 and D2 bursa. RNA from biological replicates of B cells isolated from pooled bursae (ED18 *n* = 4; D2 *n* = 3) was analyzed by qRT-PCR for CXCL12 and CXCR4 expression in comparison to RNA from total bursa RNA (ED18 *n* = 4; D2 *n* = 3). **(A,B)** Shown are mean and *SD*. Significance was calculated by One-Way-ANOVA and Bonferroni *post-hoc* test with ^*^p ≤ 0.05, ^**^p ≤ 0.01, and ^***^p ≤ 0.001 and refers in **(A)** within the analysis of CXCL12 or CXCR4 to the preceding time point and in **(B)** to the respective ED18 bursa value.

To examine if CXCL12 and CXCR4 play a role in chicken B cell development, we started with an analysis of CXCL12 and CXCR4 mRNA abundance in bursa samples from different developmental time points: ED10 = immigration of prebursal B cells, ED18 = no immigration/no emigration/strong proliferation, D2 = emigration and proliferation, D28 = terminally differentiated tissue with follicles separated in cortex and medulla. As shown in Figure 1A, the highest amount of CXCL12 mRNA was detected on ED10 with a 3-fold reduction between ED10 and ED18 and a further 50% reduction (one CT) by D2. The opposite profile was found for CXCR4 with the lowest abundance on ED10, an almost 15-fold increase between ED10 and ED18, and a further continuous increase (19- and 33-fold) at D2 and D28. Sort-purified B cells from ED18 and D2 were compared with total bursal tissue and, while CXCL12 mRNA was detected at much higher levels in bursal tissue than in B cells (500 fold at ED18 and more than 15,000 fold at D2), abundance of CXCR4 was relatively similar in B cells and total tissue (Figure 1B), which argues for CXCL12 expression by non-B-cells/stroma cells and a main expression of CXCR4 mRNA in B cells.

**Figure 2:**
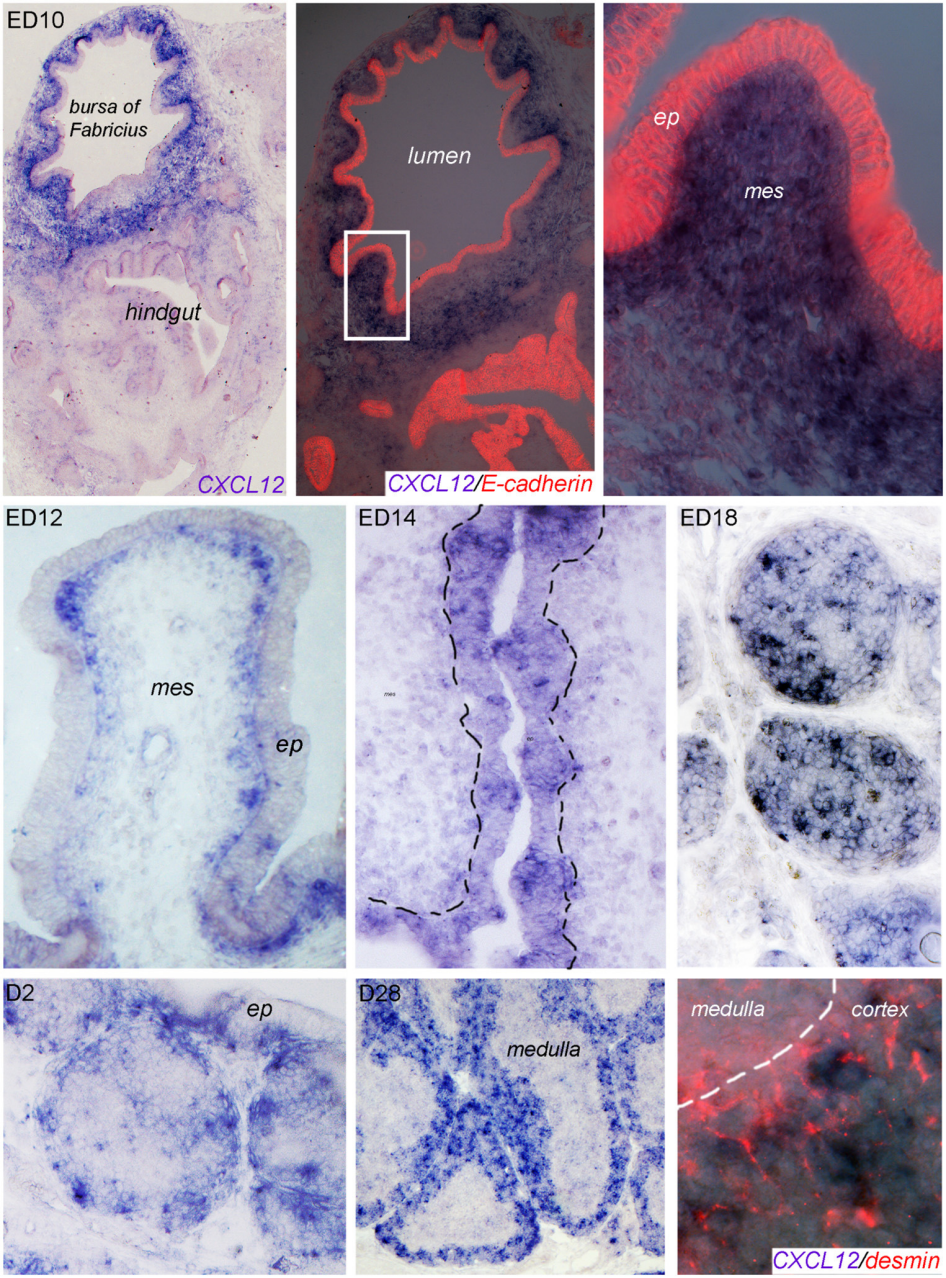
Localization of CXCL12 expression during bursal ontogeny. At the indicated time points bursa sections were analyzed by *in situ* hybridization for CXCL12 or immunocytochemistry for E-cadherin (epithelial cells) or desmin (cortico-medullary border and cortical mesenchymal cells).

*In situ* hybridization of bursal sections for CXCL12 expression shows a disseminated expression in the mesenchymal (E-cadherin negative) part of the bursa anlage at ED10 (Figure 2, upper row). At later times (ED12, ED14), the signal becomes more condensed and on ED14, CXCL12 expression can be clearly detected in the developing follicle buds. On ED18, mesenchymal expression almost disappears and a strong CXCL12 signal is spread all over the proliferating B cell follicles (Figure 2, middle row). Post-hatch, when B cell follicles start to separate into cortex and medulla, CXCL12 signal becomes more condensed at the outer part of the follicles, and in the mature bursa at D28, the expression is largely restricted to the (desmin positive) follicle cortex (Figure 2; lower row).

As CSF1R^+^ BSDCs precede B cells in the developing follicles, we wanted to know whether they may serve as source for CXCL12. Indeed, double staining of *in situ* hybridization from ED18 with the BSDC specific marker p75NTR revealed that at late embryogenesis (ED18) the CSF1R^+^/p75NTR^+^ BSDCs precursors express CXCL12. Beside BSDC precursors, we detected additional CXCL12^+^ cells. These cells are not yet identified, but the missing co-localization with the epithelial marker E-cadherin showed that they are not from epithelial origin (see Supplementary Figure 1). Interestingly, intra-follicular CXCL12 expression by BSDCs is just a transient process in the developing bursa, as post-hatch BSDCs are solely located in the follicle medulla (36) and CXCL12 expression is restricted to the cortex.

The observation that CXCL12 expression pattern is restricted and varies over time, led us to the hypothesis that CXCL12 is involved in B cell immigration into the bursa anlage (first migration step) and into the follicle bud (second migration step) and later on supports follicle separation in cortex and medulla (third migration step).

**Figure 3:**
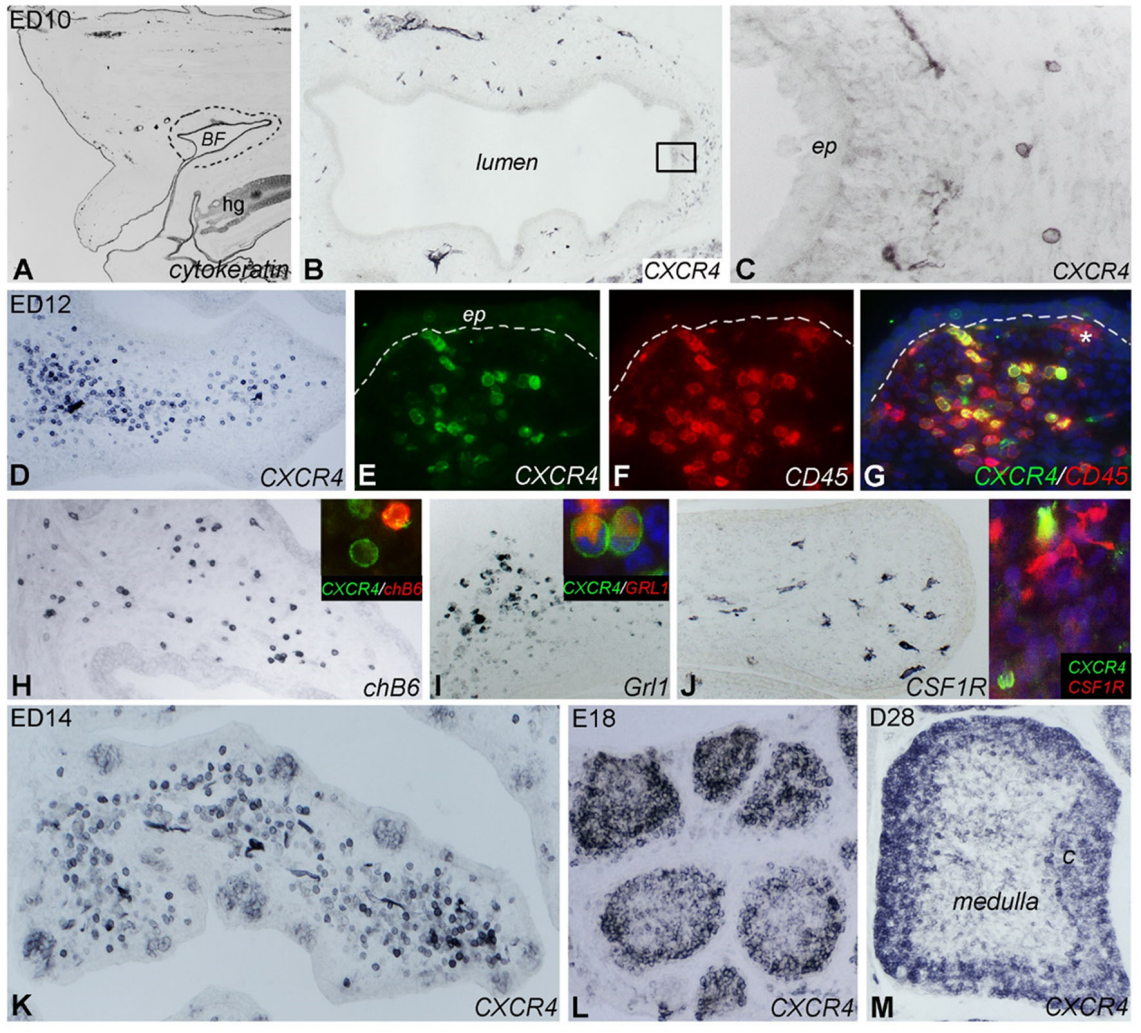
Localization of CXCR4 expression during bursal ontogeny. Immunocytochemistry of embryonic day (ED) 10 **(A–C)**, ED12 **(D–J)**, ED14 **(K)**, ED18 **(L)**, and 4 weeks old (ED28; **M**) chicken bursa of Fabricius. **(A)** The cytokeratin+ bursal epithelial anlage is a vesicle-like structure surrounded by the tail bud mesenchyme (dashed line). **(B)** A few round shaped CXCR4+ cells appear around the epithelial anlage. **(C)** Magnified image of the boxed area in **(B)**. **(D)** Bursal folds are filled with CXCR4+ cells. **(E–G)** CD45 immunostaining confirms the hematopoietic origin of CXCR4+ cells whereas the ramified CD45+ cells do not express CXCR4 (^*^ in **G**). chB6+ B cells **(H)** and Grl1+ granulocytes **(I)** are dispersed throughout the bursal fold and coexpress CXCR4 antigen (insets). **(J)** Ramified CSF1R+ cells are scattered throughout the bursa mesenchyme. Inset: double immunofluorescence staining shows that these cells do not express CXCR4. **(K)** ED14: CXCR4+ cells heavily infiltrate the bursa mesenchyme and colonize the follicle buds. **(L)** ED18: CXCR4+ cells are grouped in the lymphoid follicles. **(M)** After hatching only cortical B cells express CXCR4 antigen.

To do so, the receptor for CXCL12 must be expressed on the respective B cell populations. Therefore, we examined distribution of CXCR4 expression by immunohistochemistry with our chicken CXCR4- specific monoclonal antibody. We found that first CXCR4^+^ cells appear around E10 in the bursa mesenchyme (Figures 3A–C). From ED12 onwards, more and more cells express the CXCR4 antigen, all of them with round morphology (Figures 3D–J). While the vast majority of these cells is still located in the mesenchyme, at ED14 a distinct population is also clearly visible in the developing B cell follicles (Figure 3K).

Follicle formation and immigration of B cells are preceded by the immigration of CD45^+^/CSF1R^+^ precursors of bursal dendritic cells into follicle buds (14, 37). CD45 staining at E12 revealed that CXCR4^+^ cells are also positive for CD45 and hence of hematopoietic origin. In addition, there is a CD45^+^/CXCR4^−^ cell population in which some CSF1R^+^ ramified cells were identified (Figures 3G,J). Double-staining with the B-cell marker chB6 showed that all B cells express CXCR4 but interestingly, many CXCR4^+^ cells were chB6^−^ (Figure 3H). As the embryonic bursa is also the site of granulopoiesis (see Supplementary Figure 2 for granulocyte content at different timepoints), ED12 bursa sections were stained with the granulocyte marker Grl-1. This revealed many Grl-1^+^ cells in the mesenchyme. Double staining with Grl-1 and anti-CXCR4 showed that all granulocytes express CXCR4 (Figure 3I).

At ED14, CXCR4^+^ cells are found in the mesenchyme and in the developing follicles (Figure 3K). At ED18, CXCR4 immunoreactivity is mostly found in B cell follicles and is quite homogenous (Figure 3L), while in the mature D28 bursa, strong CXCR4 expression is found on B cells in the cortex and a much weaker staining on cells in the medulla (Figure 3M). Identical distribution as that observed for CXCR4 protein was found for CXCR4 mRNA when analyzed by *in situ* hybridization (see Supplementary Figure 3).

Taken together, during bursa ontogeny, CXCR4 is expressed on granulocytes and B cells, and its expression in follicle buds, proliferating follicles, and the mature follicle cortex parallels the presence of its ligand CXCL12.

### Changes in CXCR4 Surface Expression During B Cell Ontogeny

**Figure 4:**
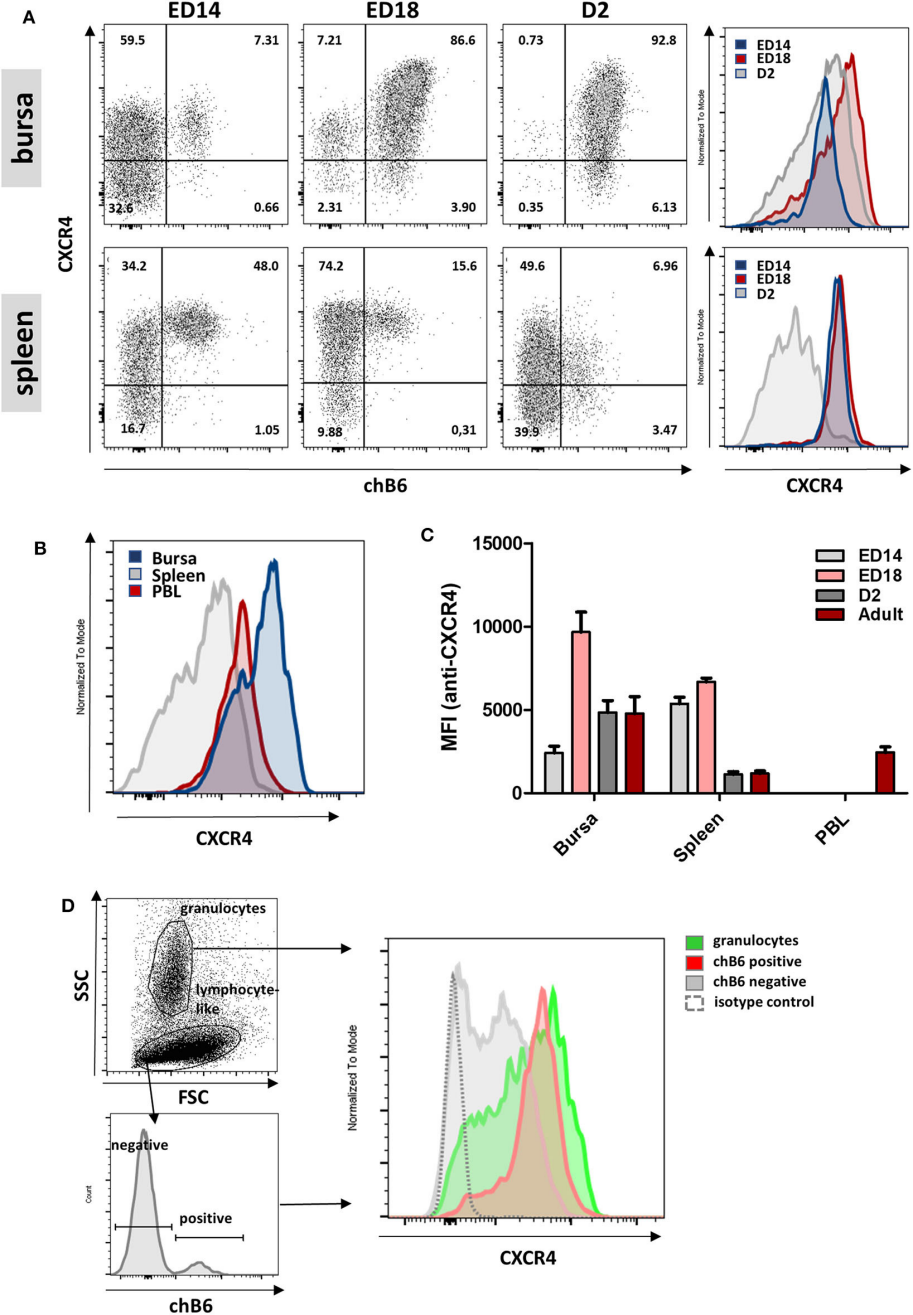
Varying CXCR4 surface expression during B cell ontogeny. **(A)** Lymphocytes were isolated from bursa and spleen at the indicated time points and analyzed for CXCR4 and chB6 expression by flow cytometry. Histograms were gated for chB6 positive cells. **(B)** CXCR4 expression on chB6 positive cells of a 4 weeks old bird. **(C)** MFI for anti-CXCR4 staining on chB6 positive cells. **(D)** Viable (7-AAD negative) cells from a ED14 bursa were separated into granulocytes and lymphocyte-like cells according to scatter properties; lymphocyte-like cells were further divided into chB6 positive and negative cells and CXCR4 expression was analyzed. **(A,B,D)** one representative of three independent experiments, **(C)** *n* = 3, mean ± *SD*.

Pre-bursal B cells migrate from the embryonic spleen to the bursa and after hatch migrate from the bursa back to the spleen. To examine whether CXCR4 could be involved in these processes, CXCR4 surface expression was analyzed in more detail by flow cytometry. Double-staining for CXCR4 and chB6 at ED14, ED18, and D2 revealed that CXCR4 is expressed on all analyzed B cell stages from spleen and bursa (Figure 4A). CXCR4 expression on bursal B cells increases drastically from ED14 [mean fluorescence intensity (MFI) = 2,400] to ED18 (MFI = 10,000), decreases after hatch and evolves from a homogenous expression at ED14 to a much more heterogeneous expression profile at later timepoints (Figures 4A,C).

At ED14, CXCR4 expression on splenic B cells (representing the cells which did not migrate to the bursa) is two-fold higher than on bursal B cells (MFI 5400 vs. 2400; *p* < 0.0001). Expression in the spleen stays roughly constant to ED18 but decreases to only a fifth (MFI ca. 1100) and even below that on bursal B cells (MFI 4800) after hatch. Interestingly, when cells from bursa and spleen were compared with PBL B cells, the latter showed a very homogenous, intermediate expression level (MFI = 2,440), which exactly correlates with the CXCR4-low shoulder of bursal cells (Figure 4B).

Cell suspensions from ED14 bursa were used to compare CXCR4 expression on granulocytes and B cells (Figure 4D) and flow cytometric analysis demonstrated a nearly identical and high receptor expression on both cell types, though expression on granulocytes was less homogenous with an additional CXCR4-low granulocyte fraction. Interestingly, a large number of chB6-negative cells with a lymphocyte-like scatter profile also express low amounts of CXCR4, indicating an even broader expression of this chemokine receptor.

Since B-cells and granulocytes show a similar expression level for CXCR4 at the early timepoints of bursal colonization, this argues for a role of CXCL12 in the immigration of B cell and granulocyte precursors into the bursa anlage.

### CXCL12 Induces Signal Transduction and B Cell Migration

**Figure 5:**
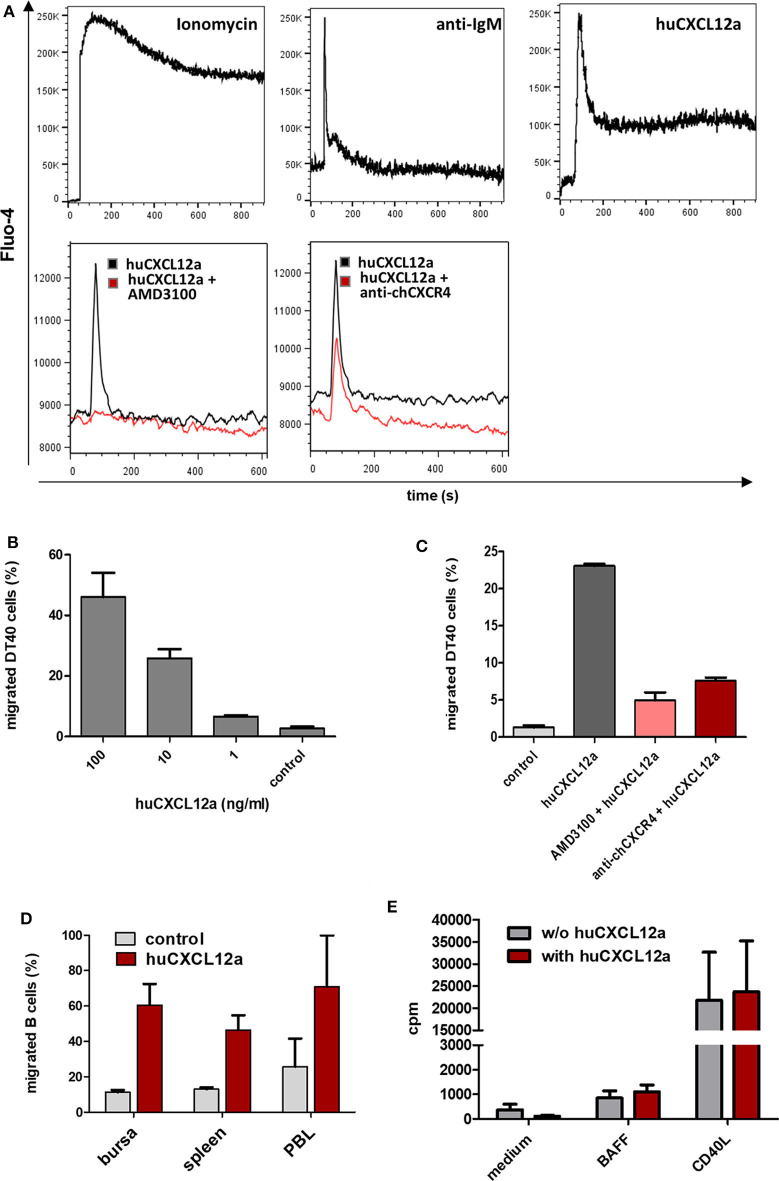
CXCR4 mediated B cell activation. **(A)** DT40 cells were loaded with Fluo-4-AM and subjected to flow cytometric calcium flux assays. After baseline recording for 60 s cells were stimulated with the indicated reagents (top row). To test potential CXCR4 inhibitors, AMD3100 (40 μM), or anti-chCXCR4 (10 μg/ml) were added first followed by huCXCL12a (100 ng/ml) 30 s later (bottom row; shown is a representative of two independent experiments). **(B,C)** DT40 were used for cell migration assays with different amounts of huCXCL12a **(B)** or 100 ng/ml huCXCL12a and the indicated CXCR4 inhibitors **(C)**. After 90 min of incubation the number of migrated cells was determined by flow cytometry (shown are mean and *SD* of triplicates from a representative experiment). **(D)** Cell suspensions from the indicated tissues were subjected to migration assays with 100 ng of huCXCL12a or control medium for 90 min before migrated cells were stained with anti-Bu1 and quantified by flow cytometry (shown are mean and *SD* of three different animals). **(E)** ^3^[H]-Thymidin uptake of isolated bursal cell, which were cultured with or without huCXCL12 (100 ng/ml) and the addition of BAFF or CD40L (mean and *SD* of three different animals).

Chemokine receptors are G-protein coupled receptors, and one of the hallmarks of their activation is mobilization of calcium from intracellular stores along with the rapid and transient rise in intracellular calcium concentrations. To test the functional interaction between CXCL12 and its receptor, we used the CXCR4-expressing bursal B cell line DT40 for calcium flux assays and cross-reactive human CXCL12 (huCXCL12) (26) protein as ligand. Binding of huCXCL12 to DT40 cells induces a rapid, strong and transient increase in intracellular Ca^2+^-levels which is similar to the signal obtained by IgM crosslinking (Figure 5A, top row). Next, we tested whether the highly specific huCXCR4-antagonist AMD3100 also binds to chCXCR4. As shown in Figure 5A (bottom row), addition of AMD3100 completely blocks the chCXCR4-mediated calcium signal. We found that the chCXCR4-specific mab is also capable of reducing the CXCL12-induced signal, though to a much lower extent.

Signaling via chemokine receptors mainly leads to cell migration. Transwell migration assays with DT40 cells demonstrated a dose-dependent migration toward huCXCL12 (Figure 5B). Addition of AMD3100 into the cell-containing insert reduced CXCL12-induced chemotaxis by more than 80%. Interestingly, the inhibitory effect of the anti-CXCR4 mab on cell migration was much stronger than on calcium mobilization as the percentage of migrated DT40 cells was reduced by about two-thirds (Figure 5C). When primary B cells from different sources were tested for their migratory capacity toward CXCL12, all analyzed B cells showed strong migration ranging from 46% of migrated splenic B cells and 61% of bursal B cells up to 71% of PBL B cells (Figure 5D). Since not only specific but also background migration was highest in PBL B cells, values for the chemotaxis index (i.e., specific migration/background migration) where very similar for B cells from PBL (3.1) and spleen (3.6) and higher for bursal B cells (5.4), although differences were not significant. In addition, we could demonstrate the effect of CXCL12 on early bursal B cells by an *ex vivo* migration assay. When complete ED13 bursae were cultured in the presence of CXCL12, large numbers of emigrating CXCR4^+^ B cells were observed (see Supplementary Figure 4).

Besides the migration-inducing potential, initial descriptions of CXCR4 function in the murine bone marrow provided indications for a growth factor function (38). Hence, we examined in [^3^H]-thymidine uptake assays, whether the addition of CXCL12 alone or in combination with survival (BAFF)- and proliferation (CD40L)-inducing cytokines can increase proliferation of isolated bursal B cells. As shown in Figure 5E, the addition of CXCL12 did not increase cell proliferation in any of the conditions.

### *In vivo* Blockade of CXCR4 Inhibits Follicle Development

**Figure 6:**
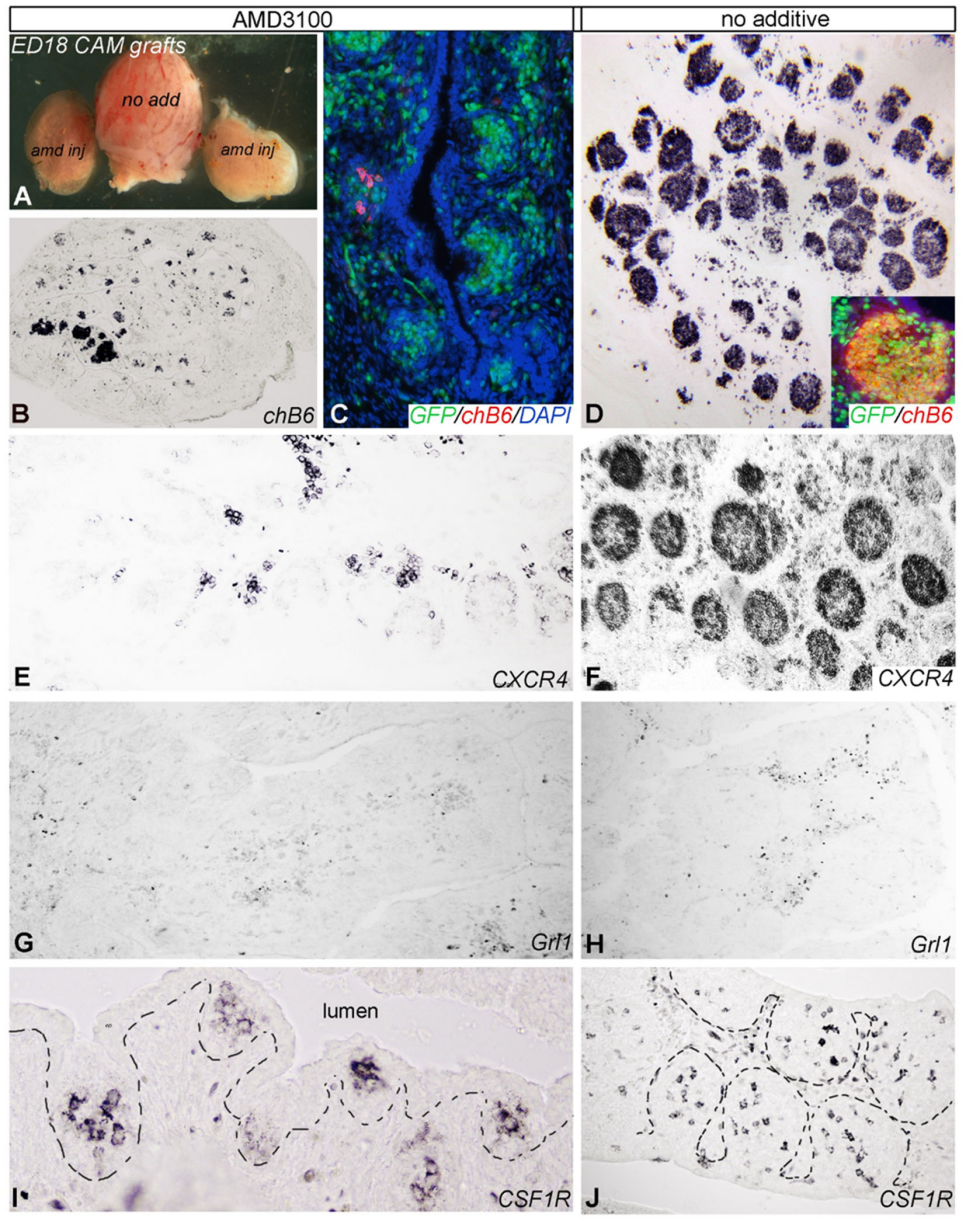
Blocking of CXCR4/CXCL12 signaling in bursa mesenchyme leads to abnormal B cell colonization. E9 bursa of Fabricius were injected with AMD3100 or vehicle (no additive) *ex vivo* and then placed onto the chorioallantoic membrane (CAM) of an E9 host GFP-chick embryo. **(A)** Comparison of bursal size of no additive and AMD3100 (amd) injected bursa of Fabricius 9 days after incubation on CAM. **(B)** Disruption of CXCR4 signaling with AMD3100 treatment resulted in reduction of size and follicle formation in the bursa. **(C,E)** Only few GPP+/chB6+/CXCR4+ B cell precursors colonized the bursa primordium cultured on CAM of age-matched GFP (green fluorescent)-chicken. **(D,F)** By contrast, non-treated bursa of Fabricius show a well-developed lymphoid follicular structure. **(G,H)** Grl1+ granulocytes scattered between follicles are present in all treatment groups. **(I,J)** CSF1R+ dendritic cell precursors colonized the surface epithelium and induced follicle bud formation.

**Figure 7:**
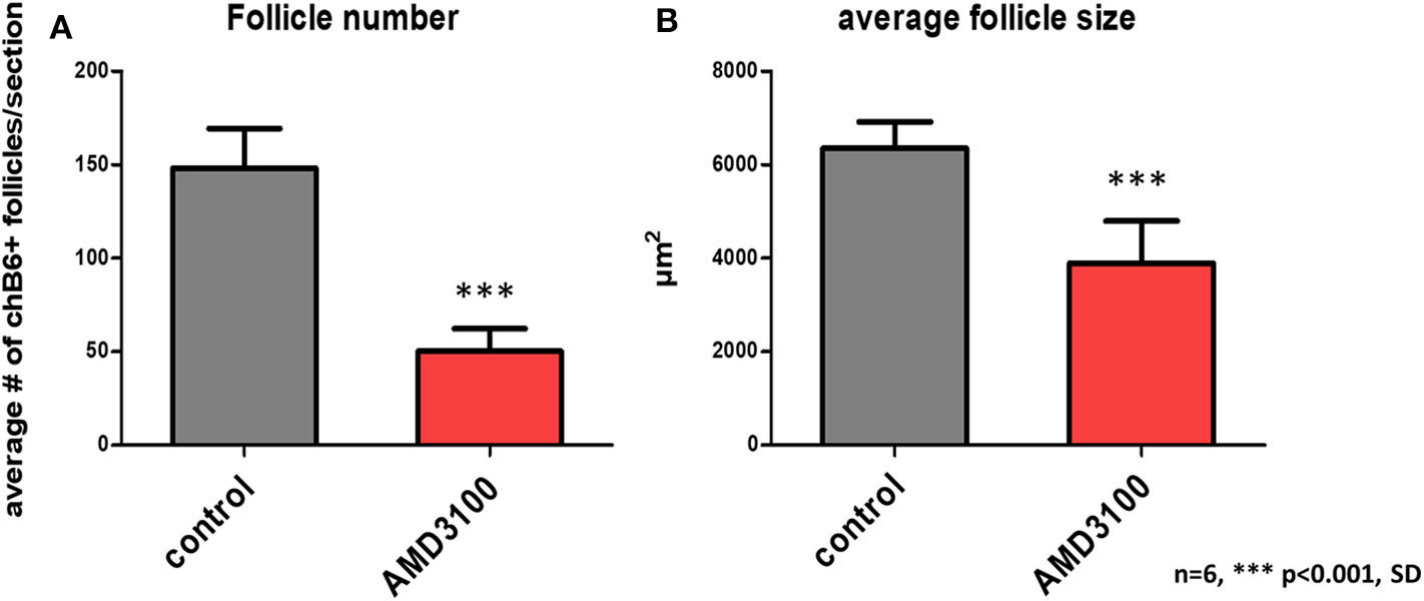
Quantification of AMD3100 *in vivo* effects. Using low magnification, size **(A)**, and number **(B)** of chB6 expressing /colonized follicles from six different AMD3100 or no additive treated bursa were determined. Because of the large number of “empty” rudimentary follicles present in AMD3100 treated bursa only structures were considered as follicle, where the cell surface epithelium-associated aggregates of chB6 cells contained at least five cells.

As Ca^2+^-signaling and chemotaxis assays had demonstrated that AMD3100 can block the interaction of CXCL12 and chicken CXCR4, we used this drug to evaluate the importance of the system *in vivo*. Therefore, bursal primordia were dissected from ED9 embryos, injected with AMD3100 or PBS and transplanted on the chorioallantoic membrane (CAM) of ED9 GFP-transgenic embryos, a timepoint which is right before the beginning of B cell immigration. After a further 9 days of incubation, the transplants were removed and analyzed by immunohistochemistry. Strikingly, AMD3100-treated bursae were much smaller than control organs (Figure 6A). B cell staining with anti-chB6 or anti-CXCR4 showed the typical structure of an ED18 bursa full of homogenous B cell follicles in controls (Figures 6D,F) while AMD3100 led to an almost complete absence of fully developed B cell follicles, with many follicles containing no B cells at all and others containing only a few scattered chB6 positive cells (Figures 6B,C,E). As the transplants were placed on CAM from GFP-expressing embryos, immigrating cells from the host could be identified by their GFP fluorescence. Immunofluorescence staining revealed that both control and treated organs were populated by many GFP positive cells, but in treated organs only few cells were chB6-positive (Figure 6C). CXCR4 staining patterns were similar to chB6 staining, with only few intrafollicular CXCR4 positive cells after AMD3100 treatment (Figure 6E). Quantitative analysis revealed a more than 60% reduction in the number of B cell follicles, with the follicles remaining in AMD3100-treated bursae showing a 40% reduction of the average follicle size (Figure 7).

Interestingly, though the number of GrL1-positive granulocytes seems slightly reduced by AMD3100 (Figures 6G,H), the effect was much weaker than on B cells. Immigration of dendritic cell precursors into the follicle bud was not affected as staining for CSF1R (a marker for bursal dendritic cells) revealed ramified immunoreactive cells in all follicle buds (Figures 6I,J).

Collectively, blocking the CXCR4–CXCL12 interaction in the bursa strongly inhibits the immigration of B cell precursors into the follicle buds and leads to a large fraction of B cell-free follicle rudiments.

### Emigrating B Cells Show Reduced CXCR4 Expression

**Figure 8:**
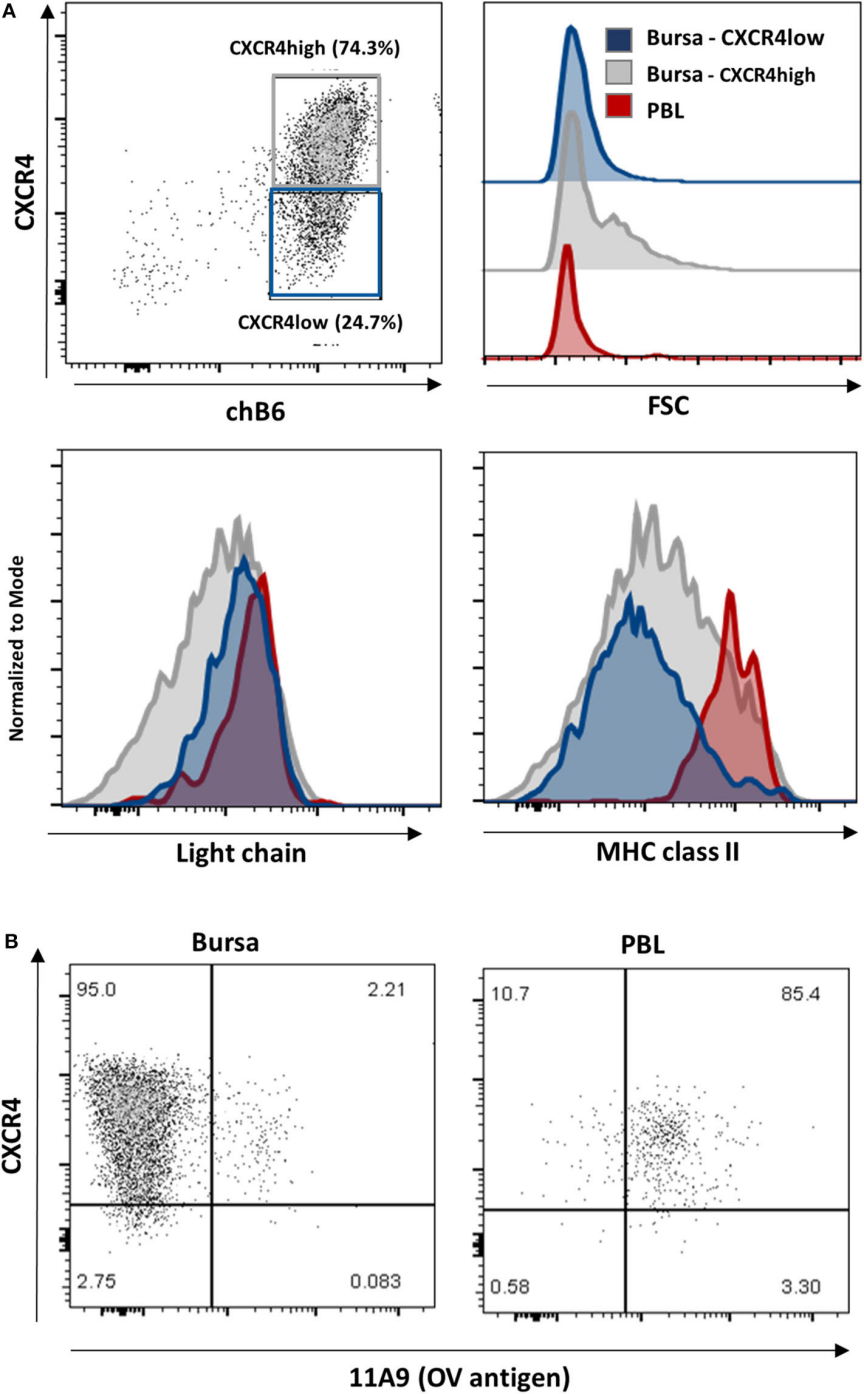
Characterization of CXCR4 low cells. Leukocytes from a 4-weeks old bursa and PBL were stained for **(A)** chB6, CXCR4, and Light chain or MHC class II or **(B)** chB6, CXCR4, and 11A9 and analyzed by flow cytometry. Histograms in **(A)** are gated for chB6+/CXCR4-low (blue) or chB6+/CXCR4-high (gray) bursal B cells and chB6+ blood B cells (red). Dot plots in **(B)** are gated for chB6+ cells.

As described above, CXCR4 expression on bursal B cells becomes heterogeneous post-hatch and allows for the separation of CXCR4-low and CXCR4-high cells, which represent about one and three quarters of the chB6^+^ cells, respectively (Figure 8A, upper left). A closer evaluation of these two populations revealed, that like PBL B cells, CXCR4-low cells have a homogenously small size, while CXCR4-high cells contain small and large cells (Figure 8A, upper right). And as on PBL B cells, homogenous, and high amounts of the BCR (measured by light chain staining) were detected on CXCR4-low cells, while anti-light chain staining on CXCR4-high cells was more heterogeneous (Figure 8A, lower left).

Ratcliffe et al. have shown that B cells which emigrate from the bursa are light chain- high, MHC class II-high and express the OV antigen (10, 39). Staining for CXCR4 and MHC class II revealed that, in contrast to MHC class II-high blood B cells, only a small part (ca. 5%) of the CXCR4-low cells in the bursa expresses high amounts of MHC class II on the surface, while CXCR4-high cells show a comparatively higher class II expression (Figure 8A, lower right). The OV-antigen, detected by the mab 11A9, was found on only 2% of bursal B cells, but the CXCR4-low phenotype of these cells was very similar to that seen in homogenous 11A9-positive blood B cells (Figure 8B).

**Figure 9:**
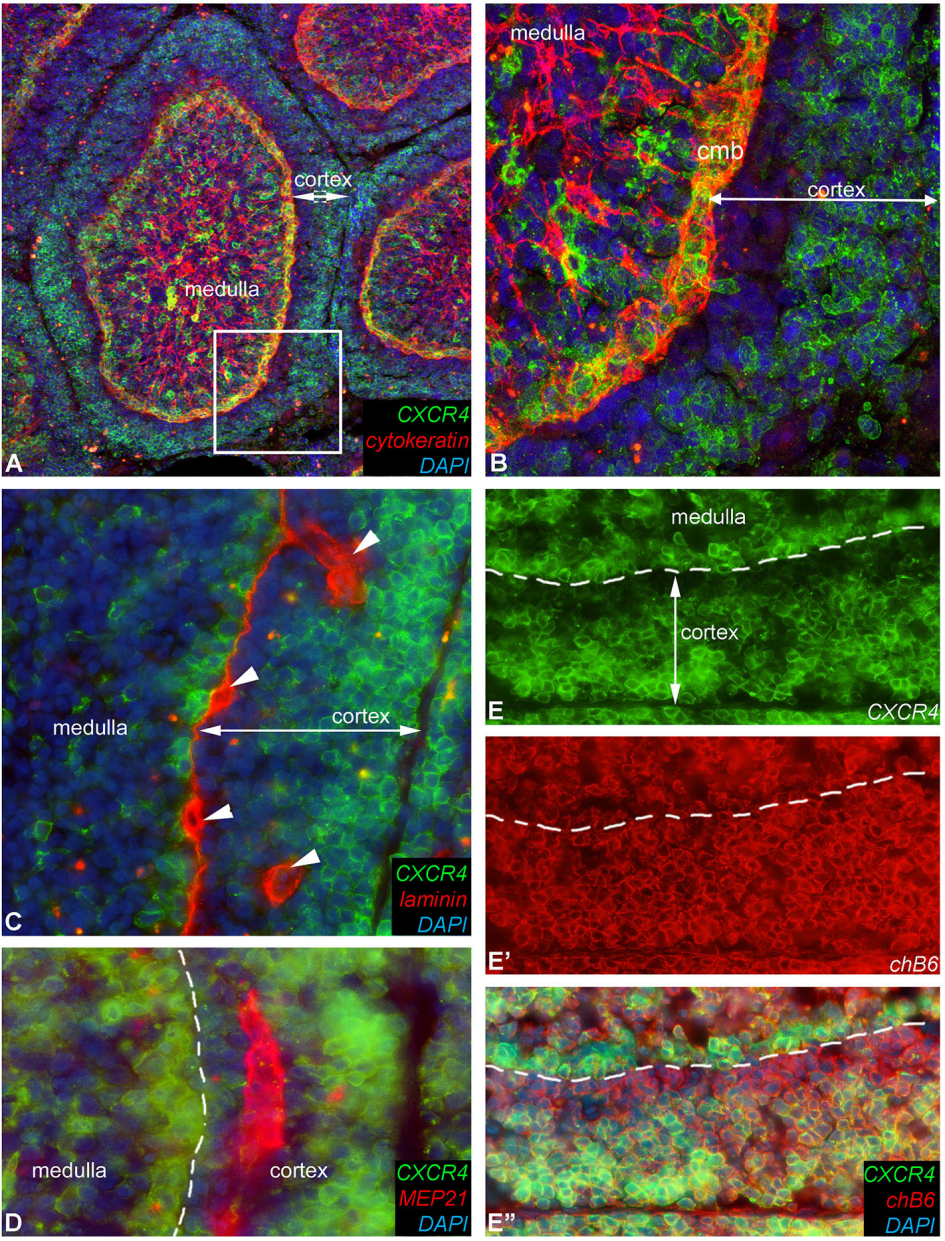
Heterogeneous CXCR4 expression in the follicle cortex. Detailed expression pattern of CXCR4 antigen in the follicle cortex. **(A)** Cytokeratin immunoreactivity labels the reticular epithelium of the medulla and the epithelium of the cortico-medullary border. **(B)** Magnified image of the boxed area in **(A)**, showing that the majority of cortical B cells are CXCR4-high but cortical cells next to the cortico-medullary border (cmb) B cells are CXCR4-low or do not express CXCR4 antigen. **(C)** Laminin mab identifies the basement membrane under the cortico-medullary border and around capillaries (arrowheads). **(D)** Mep21 is a chicken specific endothelial marker, demostrating that capillaries occur only in the CXCR4-negative cortical zone. **(E,E′,E″)** CXCR4 and chB6 positive cells are dispersed throughout the cortex but only the chB6+ B cells located in the outer region of the follicular cortex co-express CXCR4.

Strikingly, immunocytochemistry of sections from a fully mature bursa reveal a heterogeneous expression of CXCR4 on B cells in the follicular cortex with an outer broad CXCR4-high region and a narrow CXCR4-low/negative region directly adjacent to the cortico-medullary-border (Figures 9A,B,E,E′,E″). Laminin and Mep21 staining demonstrate that the capillaries through which the B cells emigrate are precisely located in this region (Figures 9C,D).

Taken together, we found that the CXCR4-low B cell population in the bursa contained light chain-high, MHC class II-high, OV^+^ small cells, which match the phenotype described for bursal emigrants and are located in the vicinity of capillaries. This suggests that B cells have to downregulate CXCR4 before they can leave the CXCL12-rich environment of the follicle cortex.

## Discussion

In large part, cell migration is guided by chemokines, and CXCL12 and its receptor CXCR4 are well-known as important contributors to central and peripheral B cell migration in mammals. As existence of chicken homologs for CXCL12/SDF-1 and CXCR4 was already described (25, 26), the aim of this project was to examine a potential role of this chemokine/chemokine receptor pair in avian B cell development.

### Bursal and Peripheral B Cells Both Respond to CXCL12

It was previously reported that receptor stimulation on chCXCR4 transfected cells and chicken PBMCs induces Ca^2+^-mobilization (25, 26). Here we show in detail that binding of crossreactive human SDF-1 strongly increases intracellular Ca^2+^ levels in CXCR4-expressing chicken cells and that the signals obtained were similar to those seen in response to IgM crosslinking and to those known from the activation of CXCR4 on mammalian cells. CXCR4 stimulation also induced strong migration of the bursal B cell line DT40, immature bursal B cells and mature blood B cells. In humans, mature and immature IgM^+^ B cells are significantly less responsive to CXCL12 than early IgM-negative B cell stages (40). For developing chicken B cells, no clear analogs to mammalian pre- and pro-B cell stages are yet defined, but it was shown that post-hatch, a very high percentage of developing bursal B cells is IgM^+^ (39). Hence, the observed similar migratory capacity of bursal and peripheral chicken B cells could correspond to mammalian data for IgM^+^ B cells, while separate experiments with pre-bursal B cells would be necessary to analyze the capacity of pro-B-cell homologs.

### B Cell Immigration Into the Bursa Anlage and the Follicle Buds Depend on CXCR4 Stimulation

B cell development in the chicken bursa of Fabricius includes several spatially and temporally distinct migratory processes. The first starts around ED10, the immigration of pre-bursal B cells into the mesenchymal part of the bursal anlage. Both qRT-PCR and *in situ* hybridization revealed a high and homogenous expression of CXCL12 in this area. As flow cytometry showed that pre-bursal B cells already express CXCR4 on the surface, CXCL12 mediated attraction of pre-bursal B cells into the bursal anlage seems highly probable.

After ED10, hematopoietic precursors of bursal secretory dendritic cells (BSDC) move from the bursal mesenchyme into the surface epithelium of the follicle folds. There, these CXCR4-negative, CSFR1^+^ cells initiate follicle bud formation by interacting with the epithelium. At that time, we observed a change in CXCL12 expression pattern. First, the homogenously distributed expression pattern developed into a more condensed signal in the sub-epithelial area of the follicle folds and at ED14 intra-epithelial CXCL12 expression in the follicle buds became prominent. In parallel, between ED12 and ED14, the early B cells migrate for the second time, crossing the basement membrane from the mesenchyme into the follicle bud. As all bursal B cells at this developmental stage are CXCR4^+^, the strong CXCL12 signal in the follicle buds most likely guides this migration. However, as only B cells immigrate into the buds but not the equally CXCR4^+^ granulocytes, it seems likely that further signals are required to initiate this second migratory step. Possible candidates therefore would be CXCL13 or CCL20 and their respective receptors CXCR5 and CCR6, as both chemokines have been described in the chicken (41) and are important for B cell migration in germinal centers and GALT structures in mice and humans (23, 42).

Blocking of CXCR4 with AMD3100 in a transplanted bursa anlage leads to a drastic reduction of B cell follicles in the ED18 bursa, demonstrating the importance of CXCL12-CXCR4 interaction for B cell immigration and follicle formation. While initial follicle induction was not affected, as demonstrated by the unaltered presence of CSF1R positive BSDCs in the follicle buds, B cell immigration into the follicles of AMD3100-treated bursa was almost completely abolished, leading to many follicle rudiments without B cells. As AMD3100 was injected in the complete ED9 bursa anlage, the experiment does not allow exact discrimination of which B cell migration step is affected, immigration of pre-bursal B cells into the bursal mesenchyme or further migration into the follicles. However, no accumulation of chB6-positive cells in the intrafollicular mesenchyme was observed in AMD3100-treated organs, strongly suggesting that CXCL12 binding is necessary for the first migration step to attract and retain pre-bursal B cells in the bursal anlage. If the CXCL12-CXCR4 axis would only be necessary for the second migration step into the follicles, one would expect a larger number of B cells in the interfollicular mesenchyme in treated organs.

As this work was being finalized for publication, a paper was published showing that adoptive transfer of pre-bursal B cells into chicken embryos resulted in reduced follicle colonization when transferred B cells were pretreated with AMD3100 (43), hence supporting our observations.

### CXCR4 Is Involved in Bursal Granulopoiesis

In addition to its role as the primary B cell organ in avian species, the embryonic bursa is also a site of granulopoiesis, with numerous granulocytes present in the bursal mesenchyme (44). We demonstrated by immunocytochemistry and flow cytometry that bursal granulocytes are CXCR4-positive. Hence, when granulocyte precursors start to seed the bursal tissue, these cells could be attracted to the bursa via CXCR4 or the developing granulocytes and / or might be kept in the tissue by the high chemokine concentration. The latter was also reported for mammalian granulopoiesis, where the expression of CXCR4 mediates retention of developing granulocytes in fetal liver and bone marrow (21). However, despite bursal granulocytes express similar amounts of CXCR4 on the surface as bursal B cells, the number of granulocytes in AMD3100-treated transplants was hardly affected. Very likely, this is not due to a lack of CXCL12-CXCR4 signaling in granulocytes, but can be explained by our experimental setting and the sequence of hematopoietic stem cell immigration into the bursa anlage. While B cells start to immigrate only around ED10 (45, 46), granulocyte precursors seed the organ earlier between ED6.5 and ED8. As the bursae for CAM transplantation were taken at ED9, there were hardly any B cells present, but probably most granulocyte precursors had already migrated into the mesenchyme. Hence, CXCL12 is probably not necessary to keep the developing granulocytes in the bursal mesenchyme, but more likely CXCR4 expression is involved in the attraction to this site of granulopoiesis. Regarding the further fate of the developing bursal heterophils, it is interesting that in humans regulated expression of CXCR4 on neutrophils is not only involved in early granulopoiesis, but also in the microbiome-/ Toll like receptor (TLR)-regulated aging of neutrophils and the homing of aged neutrophils to the bone marrow (47). Ontogeny of avian granulocytes, including the biological relevance of transient bursal granulopoiesis, is still not clear. As via the bursal duct, bursal cells are also exposed to gut derived antigen and hence, TLR-ligands (48), one could speculate that similar to mammals, in the steady state condition bursal CXCL12/CXCR4 are involved in the homeostasis of the peripheral granulocyte pool. However, as bursal granulopoiesis stops post-hatch and the microbiome does not develop before hatch, the regulation has to be different.

### CXCR4-CXCL12 Interaction Contributes to Follicle Formation and the Development of the Cortico-Medulary Architecture

Once bursal B cells have populated the follicles, they start to proliferate and diversify their BCR repertoire by gene conversion (49). In mice, CXCL12 was initially described as “pre-B cell growth stimulating factor,” which supported B cell proliferation (38). However, in spite of the high CXCL12 abundance in the bursal B cell follicles before hatching, the chemokine increased neither BAFF-mediated survival nor CD40L-induced proliferation of bursal B cells. As we used cells from post hatch bursae for those experiments, we cannot completely rule out that embryonic B cells rely on different signals than post-hatch cells and that CXCL12 does contribute to the exponential proliferation from ED14 onwards. However, CXCL12 leads only to weak stimulation in mammals, so it is unlikely to be the major proliferation-inducing factor for bursal B cells.

Interestingly, CXCR4 surface expression on ED18 bursal B cells is strongly increased as compared to ED14. Hence, it seems likely that high amounts of receptor and high CXCL12 abundance are responsible for retaining the developing B cells in the follicles, a CXCL12-CXCR4 function which is well-described for human B cells in the bone marrow (21).

Around hatch, another drastic structural change occurs in the heavily proliferating follicles. In a third migration step, some B cells migrate back across the follicle surrounding basement membrane and start to proliferate between the encircling vimentin- and desmin-expressing mesenchymal reticular cells (15, 16). Consequently, the surrounding region evolves into the follicle cortex, leading to a clear separation of the follicle into cortex and medulla. Strikingly, this results in a structure which in appearance closely resembles chicken germinal centers: globular or ovoid structures with a densely packed, highly proliferating cortex, and a less dense, less proliferating medulla, which contains macrophages and follicular dendritic cells (FDCs)/FDC-analogs (50, 51). At the same time as B cells cross the basement membrane, the pattern of CXCL12 expression changes and the signal becomes much stronger in the developing cortex than in the central follicular region (medulla). Finally, in the fully mature bursal follicle, CXCL12 mRNA was almost exclusively detected in the follicle cortex. It is well-known that in mammalian GCs sequential modulation of the CXCL12-CXCR4 axis is essential to guide B cell migration from the CXCL12-rich, highly proliferating dark zone into the CXCL12-low light zone for selection and back into the dark zone for additional rounds of proliferation and BCR mutation (23). Another lymphoid tissue where CXCL12-CXCR4 interaction probably does guide B cell migration is the rabbit appendix. Rabbits, like chickens, use a GALT structure to diversify their B cell repertoire. After the generation of a very limited BCR repertoire in the bone marrow, rabbit B cells migrate to the appendix, start to proliferate and diversify the repertoire by gene conversion and somatic hypermutation (52, 53). To do so, B cell follicles develop with a highly proliferating follicle base region and a non-proliferative apical region where FDCs are located (53) and the CXCL12-CXCR4 axis seems likely to guide the cells into the follicle base, the sole location of CXCL12 expression (54). The observation that chicken bursal B cell follicles show a CXCL12 expression pattern similar to mammalian GCs and B cell follicles in the rabbit appendix strongly argues in favor of CXCL12 guiding the third migration step of bursal B cells across the basement membrane to establish the follicle cortex/medulla architecture. Hence, we demonstrate that the localization of proliferating and diversifying B cells in a distinct follicle area by the CXCL12-CXCR4 axis is a highly conserved mechanism across species despite differing strategies of B cell development between human and murine GCs, B cell follicles in the rabbit appendix and the mature bursal follicle in chickens.

### Reduced Expression Enables Emigration of B Cells From the Bursa to the Periphery

Around hatch, the final migratory step during bursal B cell development is initiated with B cells emigrating from the bursa to seed the peripheral lymphatic structures. While in the 1st days post-hatch, only few peripheral B cells can be detected, the emigration rate increases until it reaches around 1% of PBL B cells per hour by 4 weeks post-hatch (10). At this time point the follicle cortex is fully developed and is the site where the majority of B cells emigrates (17). While CXCR4 expression on bursal B cells before hatch is high and largely homogenous, the expression level decreases post-hatch, with a small subpopulation of B cells with low CXCR4 expression that closely resembles the level of PBL B cells, (i.e., those cells that have already left the bursa). It was shown that around 5% of bursal B cells emigrate per day (12). These emigrating B cells are characterized by high surface expression of BCR and MHC class II, and positive staining for the OV antigen (10). We show here that precisely these attributes apply to a fraction of bursal CXCR4-low cells (see Table 1) and that cortical CXCR4-low B cells are located next to those capillaries enabling B cell emigration. Collectively, our data are consistent with a model where bursal B cells have to downregulate CXCR4 surface expression in order to leave the CXCL12-high environment in the follicle cortex. As this process was also described for human and mouse B cells emigrating from the bone marrow and the migration of GC B cells from the dark zone to the light zone (21–23, 55), this would represent a phylogenetically conserved mechanism.

**Table 1:** Flow cytometric characterization of different B cell populations according to CXCR4 surface expression.

	**CXCR4-high (bursa)**	**CXCR4-low (bursa)**	**PBL**
CXCR4	High	Low	Low
Size	Small and large	Small	Small
BCR	Heterogeneous	High	High
MHC class II	Heterogeneous	Low, 5% high	High
Ov-antigen	Mostly negative	2.5% positive	Positive

**Figure 10:**
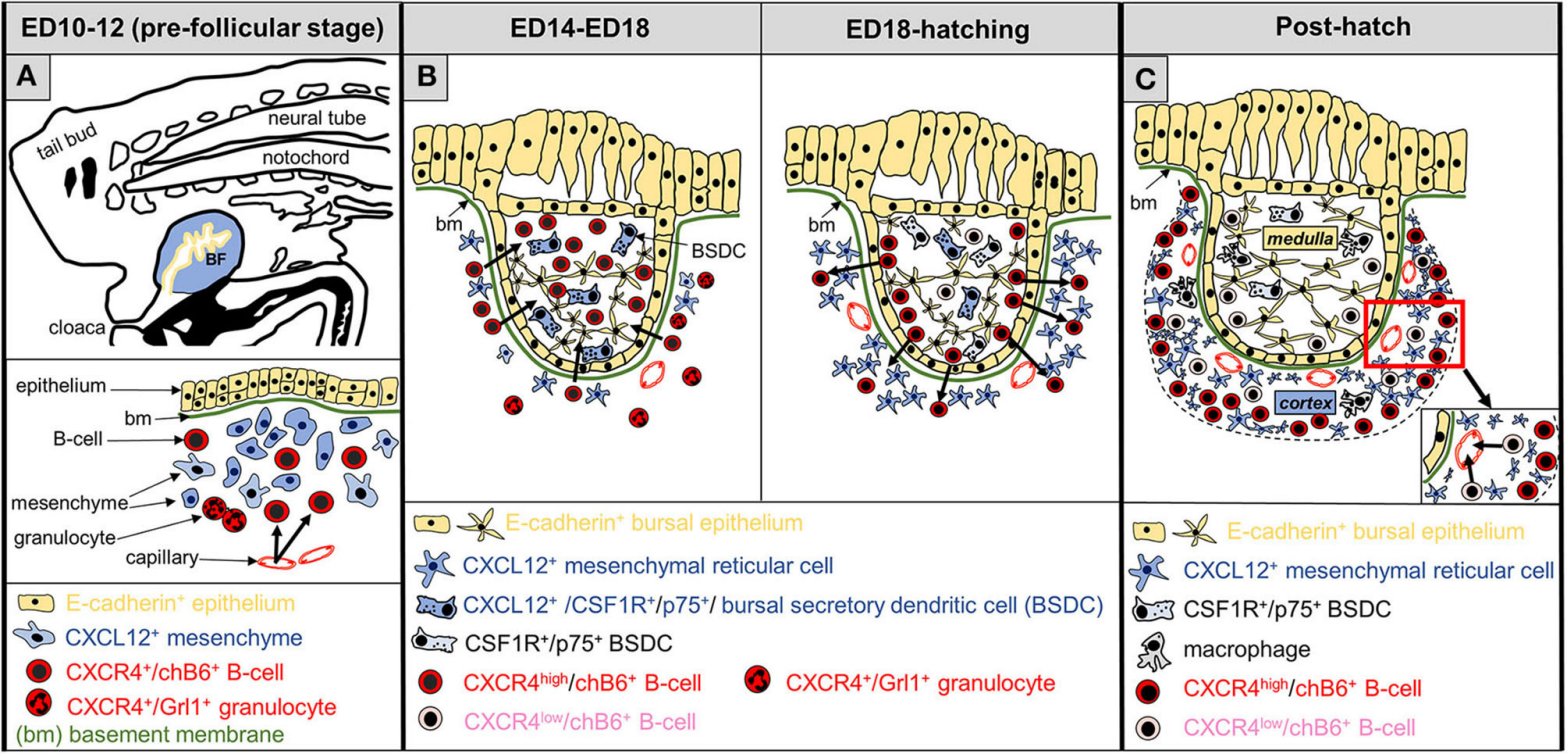
Schematic representation of CXCL12-CXCR4 functions during development of the bursa of Fabricius. **(A)** At the pre-follicular stage, B cell precursors (chB6^+^), expressing CXCR4 (red color) are attracted to the bursa anlage (arrows), where yet undefined mesenchymal cells produce CXCL12 (blue). Granulocytes, present in the bursa mesenchyme, also express CXCR4. **(B)** The follicular stage of the bursa is divided into two parts: B-cell immigration into the follicle bud (left) and development of the follicle cortex (right). Bursal secretory dendritic cells (BSDC) are the first hematopoietic cells entering the follicle buds. In the early follicular phase, CXCL12-expression pattern changes, and BSDCs become CXCL12^+^. At this stage, CXCR4^+^-B cells, but not granulocytes are attracted into the developing follicles. During the next days of bursal development, while strong intra-follicular B cell proliferation takes place (not shown), CXCL12 expression is relatively homogeneous Around hatch, CXCL12 expression declines in the follicle (BSDC lose CXCL12 expression) and increases outside of the follicle bud next to the basement membrane. At this stage, some CXCR4^+^ B cells cross the basement membrane to establish the follicular cortex, separating the follicle into a CXCL12-high cortex and a CXCL12-low medulla. **(C)** Shortly before hatch, CXCR4 expression on B cells becomes more heterogeneous and CXCR4-high and CXCR4-low B cells (pink) are distinguishable. After hatch, cortical CXCR4-high B cells are retained in the bursa, while CXCR4-low B cells are located near the cortical-medullary border and start to emigrate from the bursa via cortical blood vessels.

In summary, we have shown that CXCR4 and CXCL12 are involved in all stages of B cell migration during bursal development and are essential for the early immigration of pre-bursal B cells into the bursa anlage (see Figure 10 for an overview). In addition, our data reveal strong similarities between GCs, the rabbit appendix and the mature bursa follicle, and argue that the functions of this chemokine-chemokine receptor pair are highly conserved in evolution.

## Data Availability Statement

All datasets generated for this study are included in the article/Supplementary Material.

## Ethics Statement

Ethical review and approval was not required for the animal study because work was done in chicken embryos.

## Author Contributions

SH, NN, and BK contributed conception and design of the study. FB performed quantitative RT-PCR and flow cytometry experiments as well as migration and Ca-signaling experiments. VH and NN performed the *in situ* hybridization and embryo-manipulation experiments. NF and NN immunostained, photographed, and analyzed the adult tissues. SH wrote the first draft of the manuscript. All authors contributed to manuscript revision, read and approved the submitted version.

## Conflict of Interest

The authors declare that the research was conducted in the absence of any commercial or financial relationships that could be construed as a potential conflict of interest.
