# Supplementary figures and images for: In and Out of the Bursa—The Role of CXCR4 in Chicken B Cell Development
Source: Front Immunol. 2020 Jul 14;11:1468. doi: 10.3389/fimmu.2020.01468 (PMC7381227; doi:10.3389/fimmu.2020.01468)

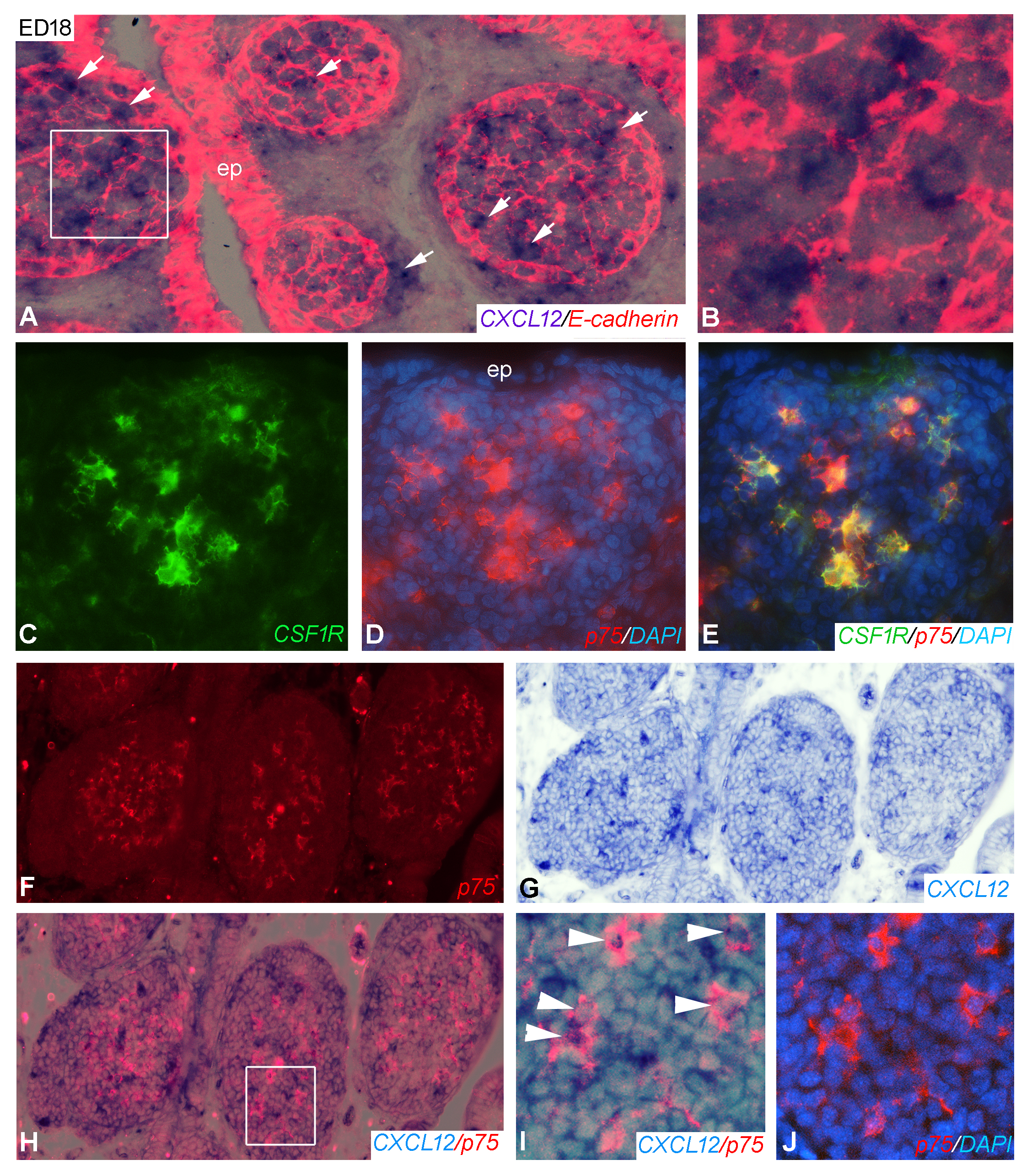

Supplement: Supplementary file 1 [file Image_1.jpg]

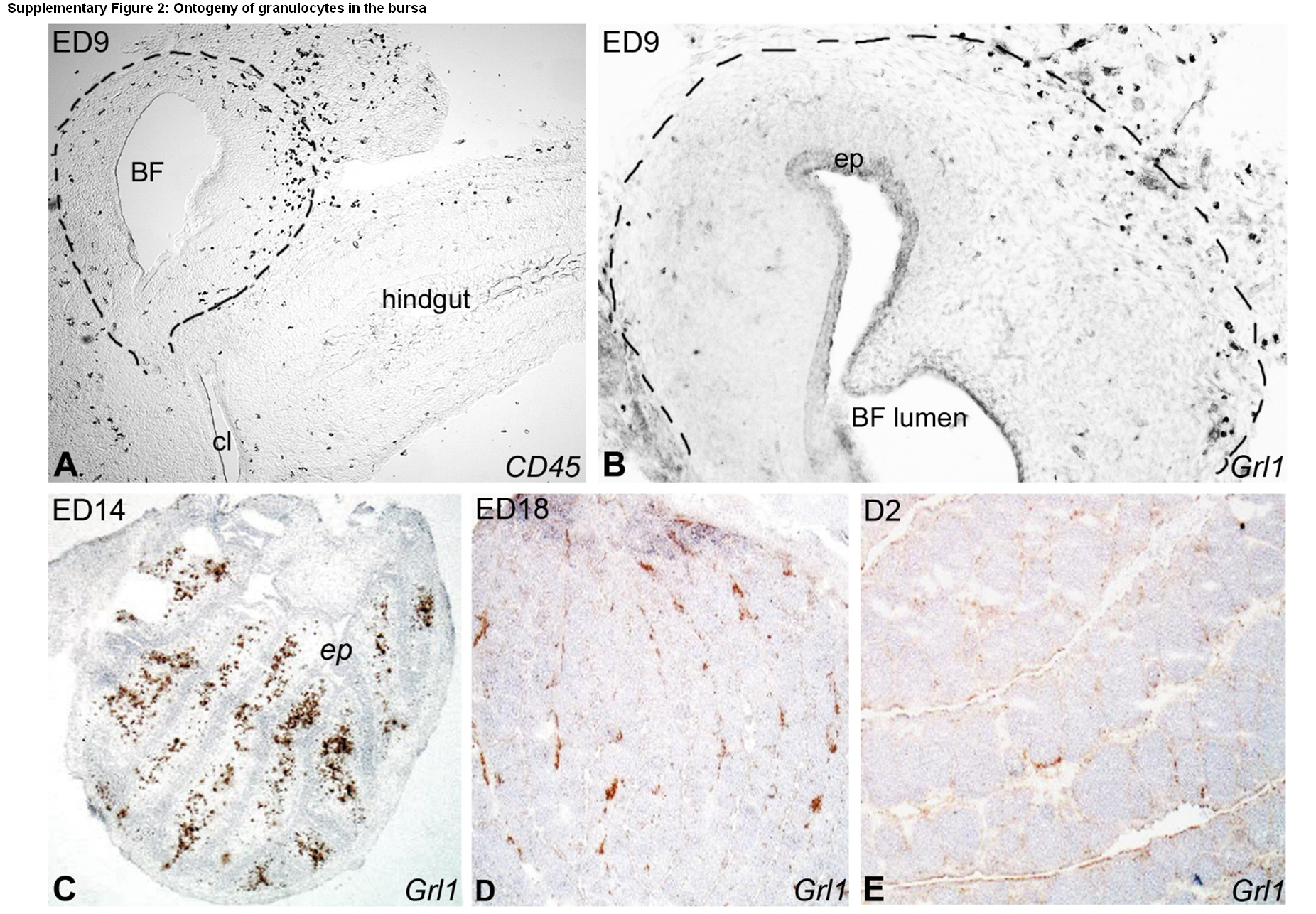

Supplement: Supplementary file 2 [file Image_2.tif]

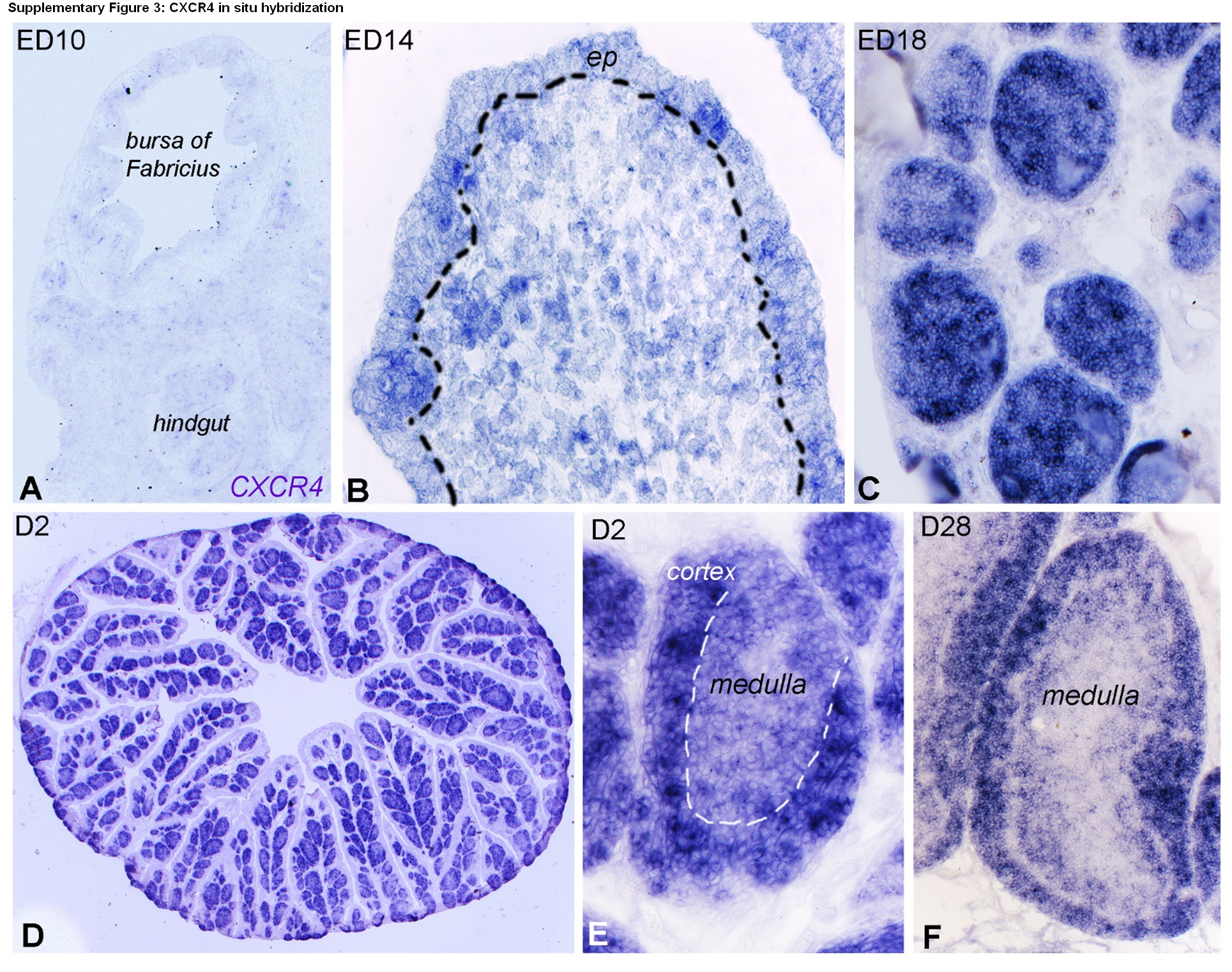

Supplement: Supplementary file 3 [file Image_3.tif]

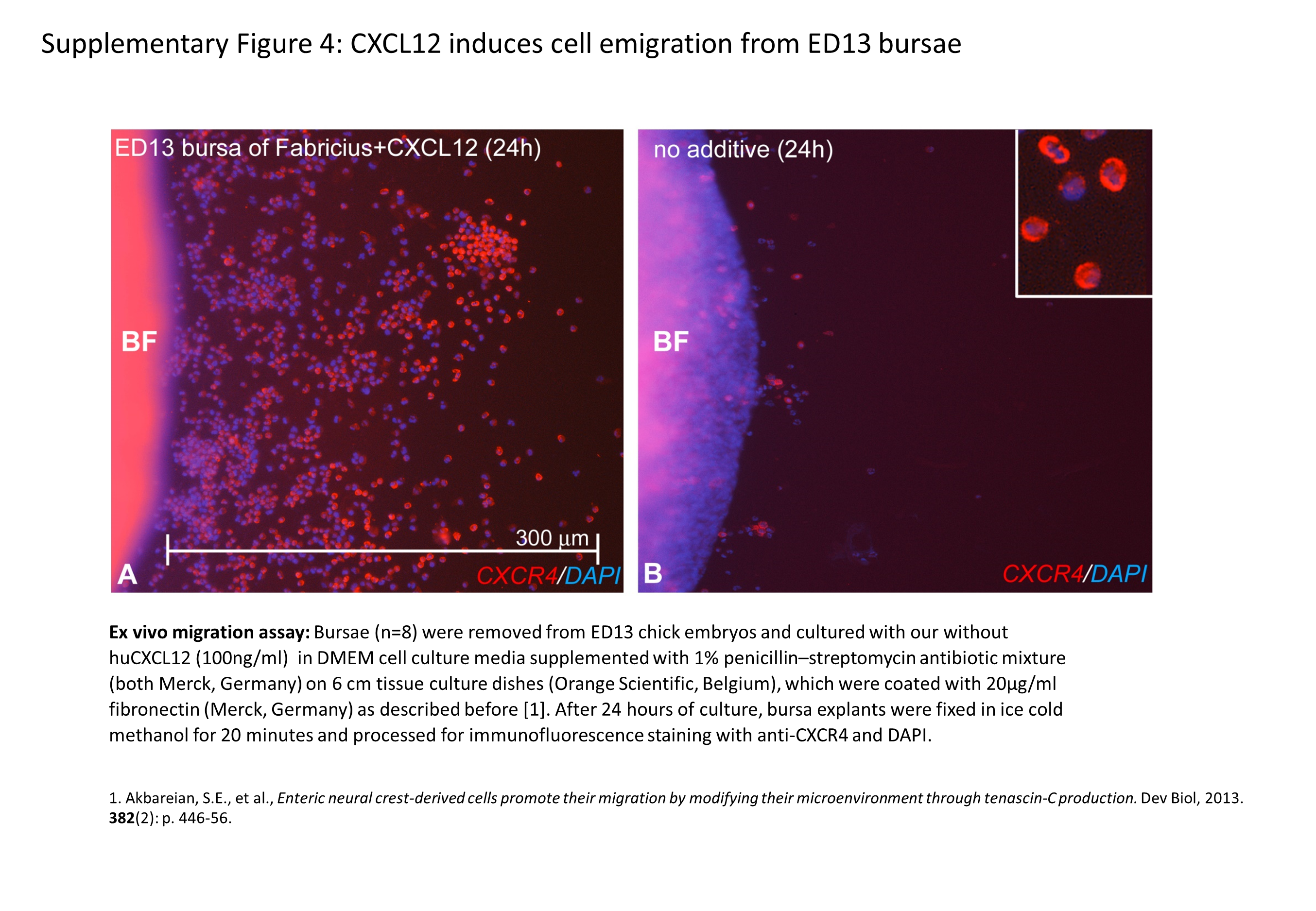

Supplement: Supplementary file 4 [file Image_4.TIF]
