# Supplementary material for: In and Out of the Bursa—The Role of CXCR4 in Chicken B Cell Development
Source: Front Immunol. 2020 Jul 14;11:1468. doi: 10.3389/fimmu.2020.01468 (PMC7381227; doi:10.3389/fimmu.2020.01468)
Supplement: Supplementary file 5 [file Table_1.DOCX]

**Supplementary Table 1: Antibodies**

| **Antigen** | **Clone** | **Isotype** | **Fluorochrome** | **Source of antibody** |
| --- | --- | --- | --- | --- |
| chB6 | AV20 | IgG1 |  | Southern Biotechnology Associates, Birmingham, USA |
| chB6 | AV20 | IgG1 | Alexa-Fluor-647 | Southern Biotechnology Associates, Birmingham, USA |
| chCXCR4 | 9D9 | IgG2a | - | [1] |
| chCD45 | HISC7 | IgG2a | - | Cedi-Diagnostics B.V. |
| cytokeratin | Basal cell cytokeratin (MAB3224) | IgG1 |  | Millipore |
| desmin | DE-R-11 | IgG1 | - | DAKO |
| E-cadherin | 36 | IgG2a | - | BD Biosciences |
| GRL-1 |  | IgG1 | - | Developmental studies hybridoma bank, Iowa city, USA |
| chLight chain | 2G1 | IgG1 | - | Bio-Rad laboratories; Hercules, USA |
| chMCH class II | 2G11 | IgG1 | - | Southern Biotechnology Associates, Birmingham, USA |
| laminin | 3H11 | IgG1 |  | Developmental Studies Hybridoma Bank, USA |
| podocalyxin | MEP21 | IgG1 | - | Kind gift of Kelly McNagny [2] |
| p75^NTR^ | rabbit polyclonal | - | - | Kind gift of Dr. Louis Reichardt, [3] |
| Ov | 11A9 | IgM | - | [4] |
| CSF1R | ROS-AV170 | IgG1 | - | Southern Biotechnology Associates, Birmingham, USA |
| Mouse-IgG1 | - | - | FITC | Southern Biotechnology Associates, Birmingham, USA |
| Mouse-IgG1 | - | - | APC | Southern Biotechnology Associates, Birmingham, USA |
| Mouse-IgG2a | - | - | PE | Southern Biotechnology Associates, Birmingham, USA |
| Mouse-IgM | - | - | FITC | Southern Biotechnology Associates, Birmingham, USA |
| Chicken-IgM | M1 | IgG1 | - | Southern Biotechnology Associates, Birmingham, USA |

1. Escot, S., et al., *Misregulation of SDF1-CXCR4 signaling impairs early cardiac neural crest cell migration leading to conotruncal defects.* Circ Res, 2013. **113**(5): p. 505-16.

2. McNagny, K.M., et al., *Thrombomucin, a novel cell surface protein that defines thrombocytes and multipotent hematopoietic progenitors.* J Cell Biol, 1997. **138**(6): p. 1395-407.

3. Weskamp, G. and L.F. Reichardt, *Evidence that biological activity of NGF is mediated through a novel subclass of high affinity receptors.* Neuron, 1991. **6**(4): p. 649-63.

4. Houssaint, E., A. Mansikka, and O. Vainio, *Early separation of B and T lymphocyte precursors in chick embryo.* J Exp Med, 1991. **174**(2): p. 397-406.
